# An Updated Meta‐Analysis of Randomized Controlled Trials on Total Hip Arthroplasty through SuperPATH versus Conventional Approaches

**DOI:** 10.1111/os.13239

**Published:** 2022-03-24

**Authors:** Nikolai Ramadanov

**Affiliations:** ^1^ Department of Emergency Medicine, University Hospital Jena Friedrich Schiller University Jena Germany; ^2^ Center of Orthopaedics and Traumatology, University Hospital Brandenburg an der Havel Brandenburg Medical School Theodor Fontane Neuruppin Germany

**Keywords:** Conventional approach, Meta‐analysis, SuperPATH, Total hip arthroplasty

## Abstract

The object was to conduct a systematic review and meta‐analysis of randomized controlled trials (RCTs) up to the present to draw reliable conclusions in the comparison between short‐term outcomes of total hip arthroplasty (THA) through supercapsular percutaneously assisted approach in THA (supercapsular percutaneously‐assisted total hip (SuperPATH)) versus conventional approaches (CAs). A systematic literature search was performed to identify RCTs comparing primary and secondary outcomes of THA through SuperPATH *vs*. CAs. Mean differences (MDs) were calculated for continuous outcomes and odds ratios (ORs) for dichotomous outcomes, using the DerSimonian and Laird method, the Mantel–Haenszel method and random effects model. A total of 14 RCTs involving 1021 patients met the inclusion criteria. Primary outcomes: SuperPATH reduced intraoperative blood loss (MD = −61.4, 95% CI −119.1 to −3.8). SuperPATH increased Harris hip score (HHS) 3, 6 and 12 months postoperatively (MD = 2.4, 95% CI 0.6–4.2; MD = 2.1, 95% CI 0.6–3.6; MD = 0.7, 95% CI 0.1–1.3; resp.). Both approaches did not differ in postoperative complication rates (OR = 0.7, 95% CI 0.2–3.3). Secondary outcomes: SuperPATH reduced pain visual analogue scale (VAS) 1 day and 3 days postoperatively (MD = −1.0, 95% CI −1.8 to −0.2; MD = −1.2, 95% CI −1.8 to −0.5; resp.). SuperPATH reduced incision length (MD = −5.2, 95% CI −7.0 to −3.4). SuperPATH increased operation time (MD = 14.3, 95% CI 3.7–24.8). Both approaches did not differ relevantly in acetabular cup inclination (MD = −1.8, 95% CI −3.8–0.2) and acetabular cup anteversion (MD = −0.6, 95% CI −1.2 to −0.1) angles. The overall findings of this meta‐analysis (Meta‐SuCAs‐2) suggested that the short‐term outcomes of THA through SuperPATH were superior to CAs. In the primary outcome, SuperPATH had a lower intraoperative blood loss and a higher HHS. Both approaches did not differ in postoperative complications. In the secondary outcome, SuperPATH had a lower pain VAS and a shorter incision length. Both approaches showed sufficient results in acetabular cup positioning. CAs had a shorter operation time than SuperPATH.

## Graphical abstract

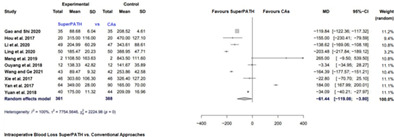

## Introduction

In the past two decades, the group of micro‐posterior approaches was introduced. In 2008 Brad Penenberg developed the percutaneously‐assisted total hip (PATH) approach^1^, a tissue‐sparing approach leading through the interval between m. gluteus medius and the conjoined tendon of the external rotators. In 2004 Stephen Murphy developed the Supercapsular (SuperCap) approach^2^, preparing the hip *in situ* to reduce soft tissue traumatization caused by the dislocation maneuver used in the conventional posterior approach. In 2011, James Chow described the supercapsular percutaneously‐assisted total hip (SuperPATH) approach, which was developed on basis of the surgical techniques of those earlier micro‐posterior approaches^3^. In this way, SuperPATH managed to combine the impressive advantages and outcomes of both micro‐posterior approaches. Important features of SuperPATH are the following: operating the hip *in situ* with the lower extremity rested on a Mayo stand during the entire operation; tissue‐sparing technique through the interval between m. gluteus medius and m. piriformis; preservation of the capsule; percutaneous accessory portal for acetabular preparation for un‐obscured visualization. Table 1 gives an overview of the conventional approaches (anterior, anterolateral, lateral transgluteal, lateral transtrochanteric, posterior, and posterolateral) to the hip joint and a more detailed description of SuperPATH.

**TABLE 1:** Overview over approaches to the hip joint

SuperPATH		Conventional approaches	
Operation steps	Descritpion	Designation	Described by:
*Position*	lateral decubitus position on a regular operating room table	*Anterior approach*	Carl Hueter (1881), Smith‐Petersen (1949), Judet (1985)
*Skin incision*	from the tip of the greater trochanter in line with the femoral axis	*Anterolateral approach*	Sayre (1884), Watson‐Jones (1936)
*Deeper preparation*	incision of the fascia of the gluteus maximus muscle	*Lateral transgluteal approach*	Bauer (1979), Hardinge (1982)
*Approach to capsule*	muscle‐sparing approach to the capsule through the space between the piriformis posterior and the gluteus minimus and medius muscle anterior	*Lateral transtrochanteric approach*	Charnley (1970)
*Further steps*	broaching proximal femur medullary canal with the reamer and implanting the prosthesis stem osteotomy the femoral neck and femoral head removal capsulotomy additional distal small incision for the reamer drive shaft and connecting it with the acetabular basket reamer through the main incision acetabular reaming, cup impaction and implantation of the inlay reposition and wound closure	*Posterior approach*	Langenbeck (1874), Kocher (1902), Gibson (1950)
		*Posterolateral approach*	Marcy and Fletcher (1954)

There are two Chinese systematic reviews and meta‐analyses, comparing the outcomes of hip arthroplasty through SuperPATH versus conventional approaches (CAs)^4^, ^5^. These studies had severe limitations. Based on the lack of quality specialist literature on this subject, further research was necessary. In 2020, Ramadanov *et al*., published the first meta‐analysis in English which compared short‐term outcomes of hip arthroplasty through SuperPATH *vs* CAs^6^. Unfortunately, the overall results still did not allow reliable conclusions. Since the publication of the last meta‐analysis on total hip arthroplasty (THA) through SuperPATH^6^, several new randomized controlled trials (RCTs) appeared in 2020 and 2021. In the meantime, a further meta‐analysis on SuperPATH was recently published. This 2021 English meta‐analysis by Ge *et al*.^7^ is an important contribution to specialist literature on SuperPATH, albeit with relevant limitations.

The following population, intervention, control, and outcomes (PICO) question was formulated: in human participants with hip disease or fracture, is the THA through SuperPATH superior compared with THA through CAs in short‐term outcomes? The present systematic review and meta‐analysis (Meta‐SuCAs‐2) of RCTs is the necessary update of the first English meta‐analysis^6^ on this topic.

## Methods

### Protocol Registration and Search Strategy

Preferred reporting items for systematic reviews and meta‐analysis‐protocols (PRISMA‐P) guidelines were followed. Since this meta‐analysis (Meta‐SuCAs‐2) was an update and continuation of the first one on hip arthroplasty through SuperPATH versus CAs^6^, using similar methodology, the present study adhered to the same review protocol, registered with the International Prospective Register of Systematic Reviews (PROSPERO) on 22 March 2020 and finally approved on 28 April 2020 (CRD42020175859) at http://www.crd.york.ac.uk/PROSPERO/. A BOOLEAN search strategy was built and adapted to the syntax of the used databases. Results of the searches were exported to a reference management software. The search continued up to February 20, 2021, using the following mesh terms and free terms: (i) PubMed: ((SuperPATH) OR (SupercapsularPercutaneously‐Assisted Total Hip)) ti,ab.; (ii) Cochrane Library: ((SuperPATH) OR (Supercapsular Percutaneously‐Assisted Total Hip)) in Title Abstract Keyword; (iii) Clinical Trials: (SuperPATH) OR (Supercapsular Percutaneously‐Assisted Total Hip); and (iv) China National Knowledge Infrastructure (CNKI): (SuperPATH). Furthermore, citations of screened studies and Google Scholar were checked for additional records. Gray literature was not considered. No restrictions to publication date or language applied. A Chinese‐speaking colleague (KL, see acknowledgements) helped by translating Chinese articles.

### Eligibility Criteria

The process was performed in two stages. The author (NR) and a colleague (RK, see acknowledgments) screened titles and abstracts to identify articles for further consideration. The full text of the selected articles was obtained and screened again for inclusion by the author (NR) and his colleague (RK). Disagreements were resolved by consensus. Kappa coefficient was used to measure the agreement between the reviewers.

Studies were selected based on the following inclusion criteria: (i) RCTs with no restriction to language and publication date; (ii) studies which compared outcomes in THA through SuperPATH and THA through CAs; and (iii) human participants with hip disease or hip fracture. RCTs with the following properties were excluded: (i) studies comparing outcomes in THA through DAA and THA through mini‐incision approaches; and (ii) surgical techniques using a computer navigation system.

### Data Extraction

Data on study characteristics, methods, quality assessment, on characteristics of participants, on details of the interventions, and on measured outcomes were extracted onto a standard electronic spreadsheet. In some cases relevant data were missing, so the related study was excluded in order to guarantee a high‐quality inclusion of RCTs.

### Risk of Bias and Level of Evidence Assessment

Risk of bias and level of evidence assessment were performed in accordance with the Cochrane's risk of bias 2 (RoB 2) tool^8^ and with the recommendations of the GRADE system^9^.

### Outcome Measurement

Meta‐SuCAs‐2 measured the following outcome parameters according to their importance. Primary outcomes were the intraoperative blood loss in ml, Harris hip score (HHS) in points, and postoperative complications such as hip dislocation, periprosthetic fracture, infection, deep vein thrombosis, and haematoma. Secondary outcomes were the pain visual analogue scale (VAS) in points, operation time in min, incision length in cm, acetabular cup inclination, and anteversion angles in degrees.

### Statistical Analysis

The entire statistical part of Meta‐SuCAs‐2 was conducted by the author (NR) of the study and by a professional statistician (SB, see acknowledgments),using the R packages meta^10^ and metaphor^11^. In Meta‐SuCAs‐2, SuperPATH represented the “experimental group” and CAs represented the “control group.” Both fixed and random effects models were tested. Random effects models provided more reliable results. So, mean differences (MDs) with 95% CIs were calculated for continuous outcomes, using the DerSimonian and Laird method and random effects model. Odds ratio (OR) and their 95% confidence intervals (CIs) were calculated for dichotomous outcomes, using the Mantel–Haenszel method and random effects model. An odds ratio of less than 1 favored the experimental group. Study weighting was performed by inverse variance^12^. The *t*‐test was calculated to determine statistically significant differences between the means of the two groups. A significance level of *P* = 0.05 was used. Study data that were clinically too diverse were not pooled. Heterogeneity was assessed using Cochrane's *Q* test (*p*‐value <0.10 is indicative of heterogeneity) and Higgins' test *I* ^2^ (low heterogeneity: <25%, moderate heterogeneity: 25%–75%, and high heterogeneity: >75%)^13^. The results were evaluated and analyzed on basis of the Cochrane handbook for systematic reviews of interventions^12^. Forest plots were used to graphically present the results of individual studies and the respective pooled estimate of effect size. Publication bias was tested using contour‐enhanced funnel plots.

## Results

### Study Identification and Selection

**Fig. 1:**
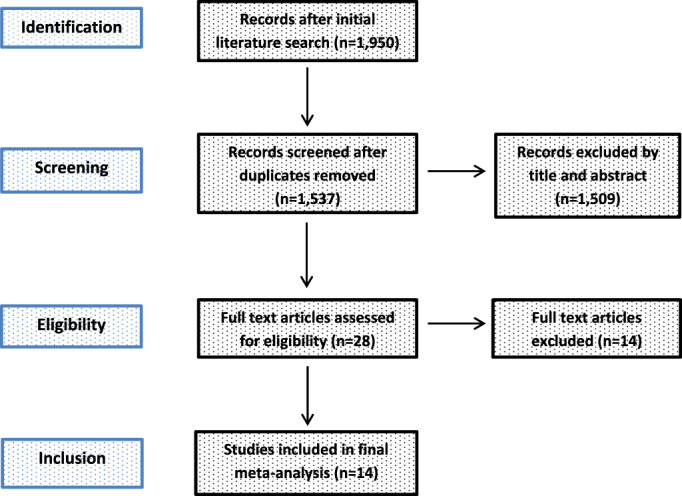
PRISMA flow diagram

A description of the study selection process is given in a PRISMA flow diagram (Fig. 1). After removing 413 duplicates, a total of 1537 studies were identified in the initial literature search. Twenty‐eight studies were assessed for eligibility after the first screening procedure by title and abstract (*κ* = 1.0) with total agreement by the author (NR) and his colleague (RK, see acknowledgments). Of these studies, 14 were excluded after the second screening procedure by full‐paper analysis (*κ* = 1.0), leaving a total of 14 studies on THA through SuperPATH versus CAs for inclusion in the final meta‐analysis^14^, ^15^, ^16^, ^17^, ^18^, ^19^, ^20^, ^21^, ^22^, ^23^, ^24^, ^25^, ^26^, ^27^.

### Characteristics of the RCTs

The 14 RCTs on THA through SuperPATH with CAs were published between 2016 and 2021, altogether involving 1021 patients (with 1044 operated hip joints). Of the included patients, 497 were operated through SuperPATH and 524 through CAs. The sample size of the studies on SuperPATH ranged from four to 154 patients. Three studies were published in English language^16^, ^19^, ^24^, eight studies were published in Chinese with an English abstract^15^, ^18^, ^20^, ^21^, ^22^, ^25^, ^26^, ^27^, and three studies were published only in Chinese^14^, ^17^, ^23^. Of the 14 studies, nine included conventional THA through posterolateral approach^16^, ^17^, ^18^, ^19^, ^20^, ^21^, ^23^, ^26^, ^27^, two through posterior approach^14^, ^24^, one through lateral transgluteal approach^25^. In two studies the surgical approach was conventional, but not further specified^15^, ^22^. The main characteristics of the patients included in the 14 RCTs were presented in Table 2. The main preoperative diagnoses were osteoarthritis, femoral neck fracture, and avascular necrosis of the femoral head.

**TABLE 2:** Main characteristics of the included RCTs

	Sample size, *n*		Surgical approach		Mean age, y (SD or range)		Gender (M/F), *n*		BMI, kg/m^2^ (SD or range)	
Study	Pts	Hips	S	CAs	S	CAs	S	CAs	S	CAs
Gao and Shi^14^	70	70	35	35 p	64.88 ± 12.13	62.18 ± 14.72	23/12	20/15	23.09 ± 2.57	23.21 ± 2.44
Hou *et al*.^15^	40	40	20	20 c	54.3 ± 13.7	53.8 ± 12.9	13/7	12/8	24.5 ± 3.6	23.9 ± 4.1
Li *et al*.^16^	96	96	49	47 pl	75.53 ± 7.34	77.21 ± 7.84	27/22	24/23	22.99 ± 2.87	22.7 ± 3
Li^17^	60	60	30	30 pl	70.35 ± 4.26	70.12 ± 4.78	16/14	18/12	–	–
Ling *et al*.^18^	100	100	50	50 pl	89.14 ± 3.6	88.95 ± 3.71	31/69	29/71	–	–
Meng *et al*.^19^	4	8	4	4 pl	51.00 ±4.54		4/0		21.49 (19.60–23.04)	
Ouyang *et al*.^20^	24	24	12	12 pl	54 (45–71)	55 (47–67)	8/4	9/3	23.1 (17.5–26.7)	23.9 (16.9–30.4)
Pan *et al*.^21^	116	116	58	58 pl	65.23 ± 6.84	65.62 ± 6.96	34/82	33/83	22.24 ± 4.15	22.56 ± 4.22
Ren *et al*.^22^	42	42	21	21 c	57.96 ± 6.89	58.45 ± 6.25	12/9	13/8	–	–
Wang and Ge^23^	95	95	43	42 pl	71.53 ± 3.76	71.58 ± 3.79	26/17	24/18	22.47 ± 1.12	22,51 ± 1,15
Xie *et al*.^24^	92	92	46	46 p	66.6 ± 11.88	64.47 ± 12.09	12/34	19/27	23.62 ± 1.63	24.06 ± 2.72
Yan *et al*.^25^	154	173	70	103 l	66 (59–75)	65 (56–82)	29/35	42/48	24.5 (17.3–31.1)	23.6 (18–32.3)
Yuan *et al*.^26^	84	84	40	44 pl	74.3 (67–79)	75.7 (69–82)	24/16	21/23	22.73 ± 1.71	22.36 ± 1.89
Zhang *et al*.^27^	54	54	27	27 pl	62.41 ± 6.44	61.28 ± 6.7	10/17	12/15	24.53 ± 5.31	23.93 ± 4.89

Abbreviations: CAs, conventional approaches; c, conventional approach; l, lateral approach; p, posterior approach; pl, posterolateral approach; Pts, patients; S, SuperPATH.

### Risk of Bias and Level of Evidence

Two out of the 14 RCTs were rated with a low risk of bias^16^, ^19^, nine RCTs with a moderate risk of bias^14^, ^15^, ^20^, ^21^, ^23^, ^24^, ^25^, ^26^, ^27^, and three RCTs with a high risk of bias^17^, ^18^, ^22^ (Table 3). Table 4 shows the level of evidence assessment according to GRADE recommendations (Table 4).

**TABLE 3:** Risk of bias assessment

Study	Random sequence generation	Allocation concealment	Blinding	Complete outcome data	Selective reporting	Other sources of bias	Overall risk of bias
Gao and Shi^14^	Y	U	U	Y	Y	Y	Moderate RB
Hou *et al*.^15^	Y	U	U	Y	Y	Y	Moderate RB
Li *et al*.^16^	Y	Y	Y	Y	Y	Y	Low RB
Li^17^	Y	U	U	N	Y	Y	High RB
Ling *et al*.^18^	Y	Y	U	N	Y	Y	High RB
Meng *et al*.^19^	Y	Y	Y	Y	Y	Y	Low RB
Ouyang *et al*.^20^	Y	Y	U	Y	Y	Y	Moderate RB
Pan *et al*.^21^	Y	Y	U	Y	Y	Y	Moderate RB
Ren *et al*.^22^	Y	U	U	N	Y	Y	Low RB
Wang and Ge^23^	Y	U	U	Y	Y	Y	Moderate RB
Xie *et al*.^24^	Y	Y	U	Y	Y	Y	Moderate RB
Yan *et al*.^25^	Y	U	U	Y	Y	Y	Moderate RB
Yuan *et al*.^26^	Y	U	U	Y	Y	Y	Moderate RB
Zhang *et al*.^27^	Y	Y	U	Y	Y	Y	Moderate RB

Abbreviations: N, negative result; RB, risk of bias; U, unclear; Y, positive result.

**TABLE 4:** Level of evidence assessment according to GRADE recommendations

No. of studies	Design	Risk of bias	Inconsistency	Indirectness	Imprecision	Other considerations	Quality of evidence
Intraoperative blood loss							
10	RCT	Serious	Serious	No serious indirectness	Serious	All studies were from China	Low
HHS 3 months postoperatively							
10	RCT	Serious	Serious	No serious indirectness	Serious	All studies were from China	Low
HHS 6 months postoperatively							
8	RCT	Serious	No serious inconcistency	No serious indirectness	Serious	All studies were from China	Very low
HHS 12 months postoperatively							
5	RCT	Moderate	No serious inconcistency	No serious indirectness	Serious	All studies were from China	Very low
Postoperative complications							
5	RCT	Serious	Serious	No serious indirectness	No serious imprecision	All studies were from China	Very low
Pain VAS 1 day postoperatively							
4	RCT	Moderate	No serious inconcistency	No serious indirectness	Serious	All studies were from China	Very low
Pain VAS 3 days postoperatively							
4	RCT	Moderate	No serious inconcistency	No serious indirectness	Serious	All studies were from China	Very low
Operation time							
11	RCT	Serious	Serious	No serious indirectness	Serious	All studies were from China	Low
Incision length							
11	RCT	Serious	No serious inconcistency	No serious indirectness	Serious	All studies were from China	Very low
Acetabular cup inclination angle							
5	RCT	Serious	No serious inconcistency	No serious indirectness	Serious	All studies were from China	Very low
Acetabular cup anteversion angle							
4	RCT	Moderate	Serious	No serious indirectness	Serious	All studies were from China	Low

Abbreviations: HHS, Harris hip score; RCT, randomized controlled trial; VAS, visual analogue scale.

### Outcomes

#### Primary Outcomes

##### Intraoperative Blood Loss

**Fig. 2:**
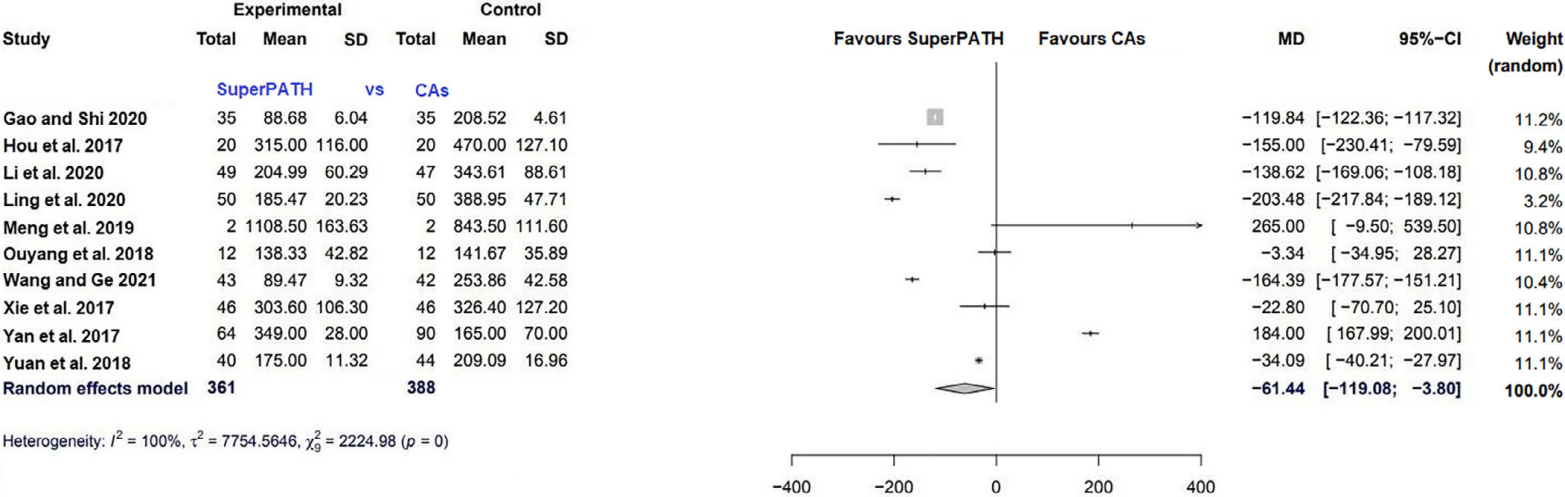
Forest plot of the primary outcome: intraoperative blood loss in ml. Abbrevuations: CAs, conventional approaches; SD, standard deviation; MD, mean difference; CI, confidence interval

Data on 749 patients were pooled from 10 RCTs (*I* ^2^ = 100%, *P* = 0, Fig. 2). The intraoperative blood loss of SuperPATH was 61.4 ml less than the intraoperative blood loss of CAs (MD = −61.4, 95% CI −119.1 to −3.8).

##### Functional Assessment

**Fig. 3:**
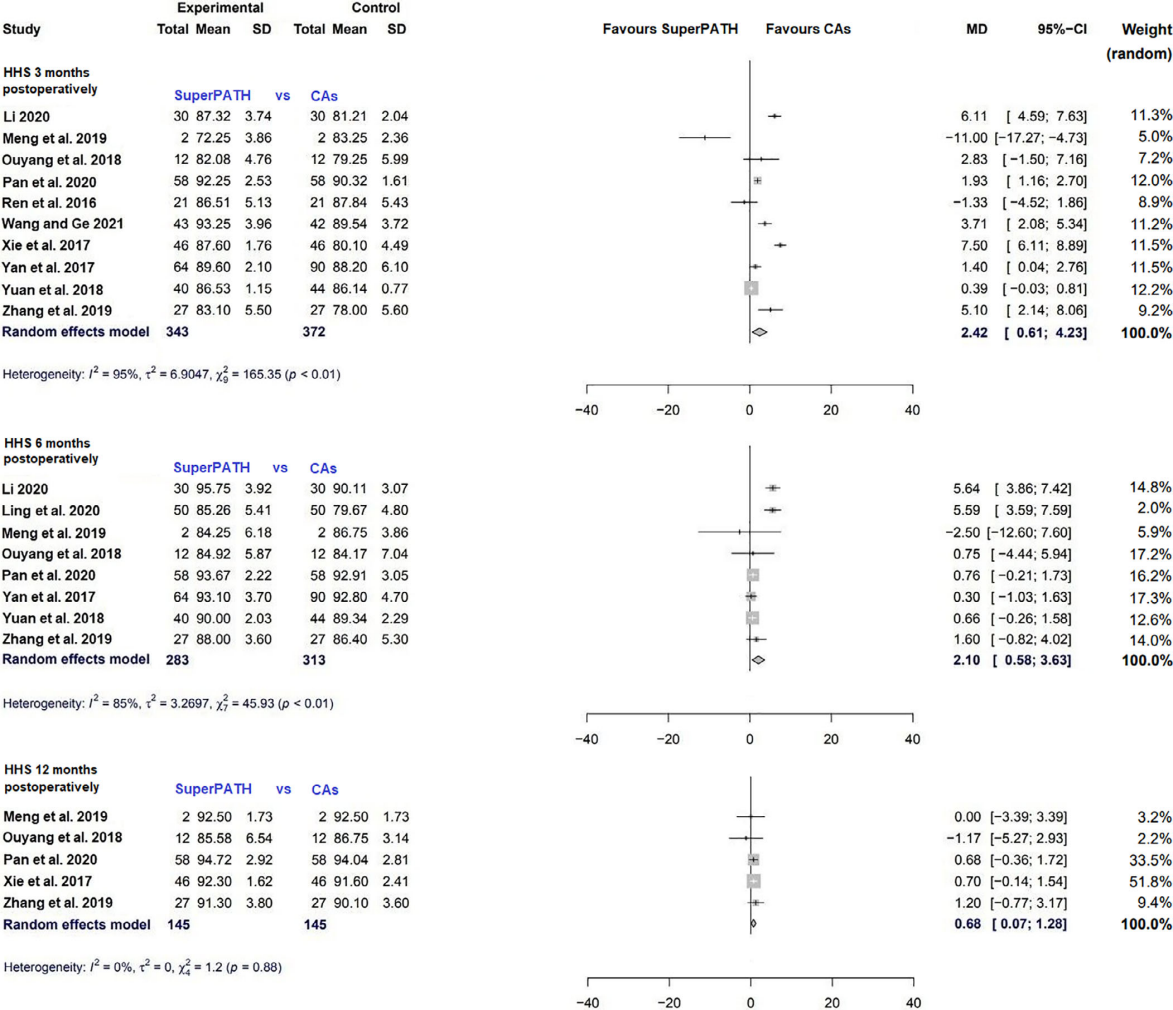
Forest plot of the primary outcome: HHS 3, 6 and 12 months postoperatively in points. CAs, conventional approaches; SD, standard deviation; MD, mean difference; CI, confidence interval; HHS, Harris hip score

Data on 715 patients were pooled from 10 RCTs (*I* ^2^ = 95%, *P* < 0.01, Fig. 3). The HHS 3 months postoperatively of SuperPATH was 2.4 points higher than the HHS 3 months postoperatively of CAs (MD = 2.4, 95% CI 0.6–4.2).

Data on 596 patients were pooled from eight RCTs (*I* ^2^ = 85%, *P* < 0.01, Fig. 3). The HHS 6 months postoperatively of SuperPATH was 2.1 points higher than the HHS 6 months postoperatively of CAs (MD = 2.1, 95% CI 0.6–3.6).

Data on 290 patients were pooled from five RCTs (*I* ^2^ = 0%, *P* = 0.88, Fig. 3). The HHS 12 months postoperatively of SuperPATH was 0.7 points higher than the HHS 12 months postoperatively of CAs (MD = 0.7, 95% CI 0.1–1.3).

##### Postoperative Complications

**Fig. 4:**
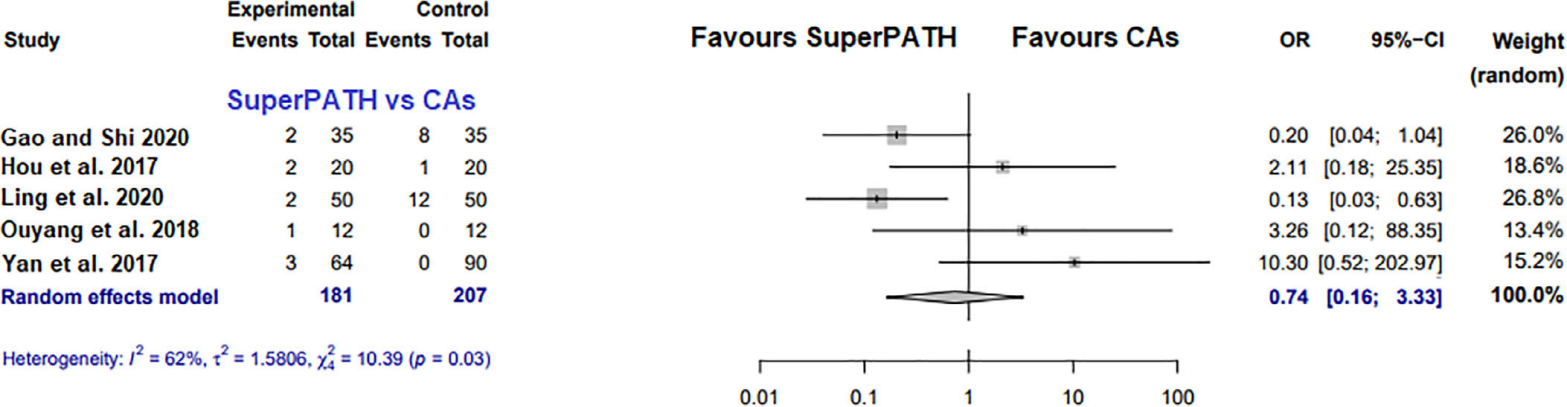
Forest plot of the primary outcome: postoperative complication rates. CAs, conventional approaches; OR, odds ratio; CI, confidence interval

Data on 388 patients were pooled from 5 RCTs (*I* ^2^ = 62%, *P* = 0.03, Fig. 4). There was no difference in postoperative complication rates (OR = 0.7, 95% CI 0.2–3.3).

#### Secondary Outcomes

##### Pain Assessment

**Fig. 5:**
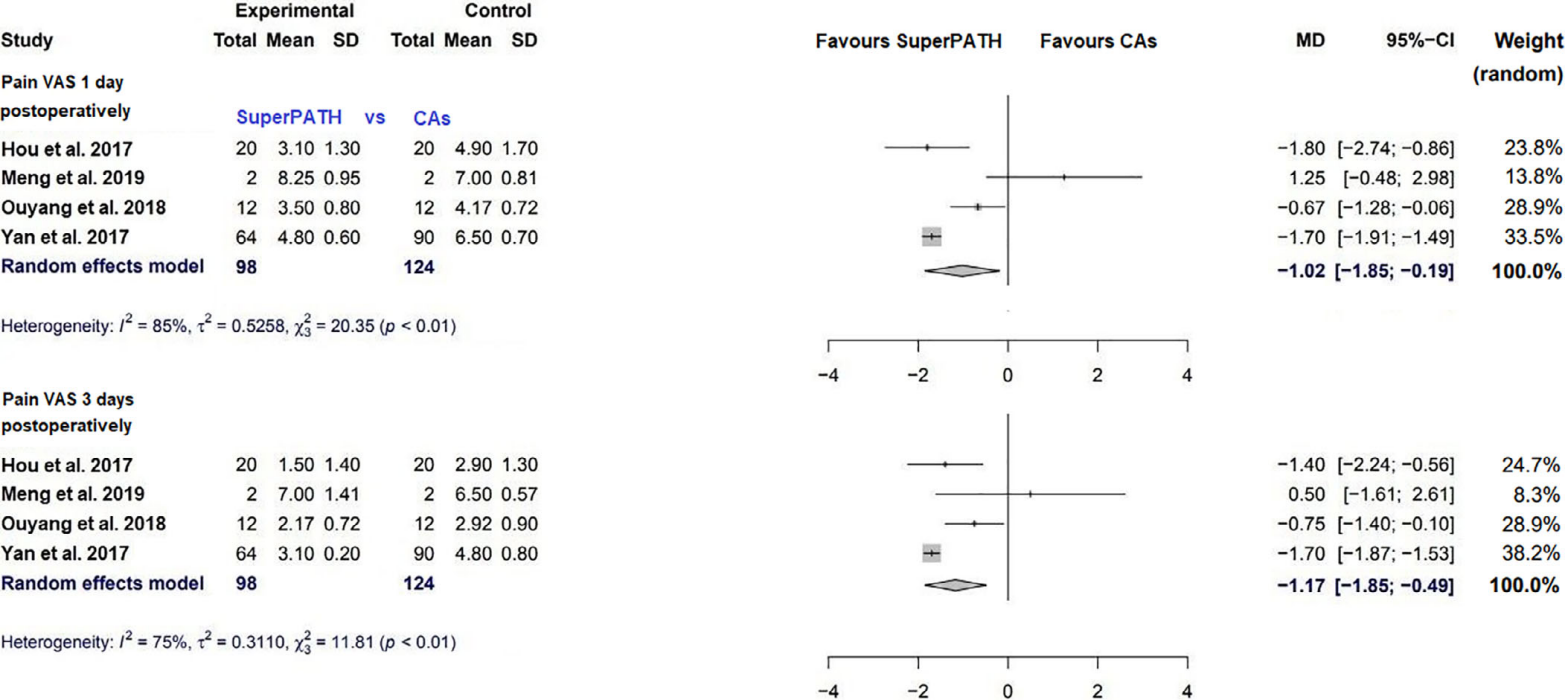
Forest plot of the secondary outcome: pain VAS 1 day and 3 days postoperatively in points. CAs, conventional approaches; SD, standard deviation; MD, mean difference; CI, confidence interval; VAS, visual analogue scale

Data on 222 patients were pooled from 4 RCTs (*I* ^2^ = 85%, *P* < 0.01, Fig. 5). The pain VAS 1 day postoperatively of SuperPATH was 1.0 points less than the pain VAS 1 day postoperatively of CAs (MD = −1.0, 95% CI −1.8 to −0.2).

Data on 222 patients were pooled from 4 RCTs (*I* ^2^ = 75%, *P* < 0.01, Fig. 5). The pain VAS 3 days postoperatively of SuperPATH was 1.2 points less than the pain VAS 3 days postoperatively of CAs (MD = −1.2, 95% CI −1.8 to −0.5).

##### Operation Time

**Fig. 6:**
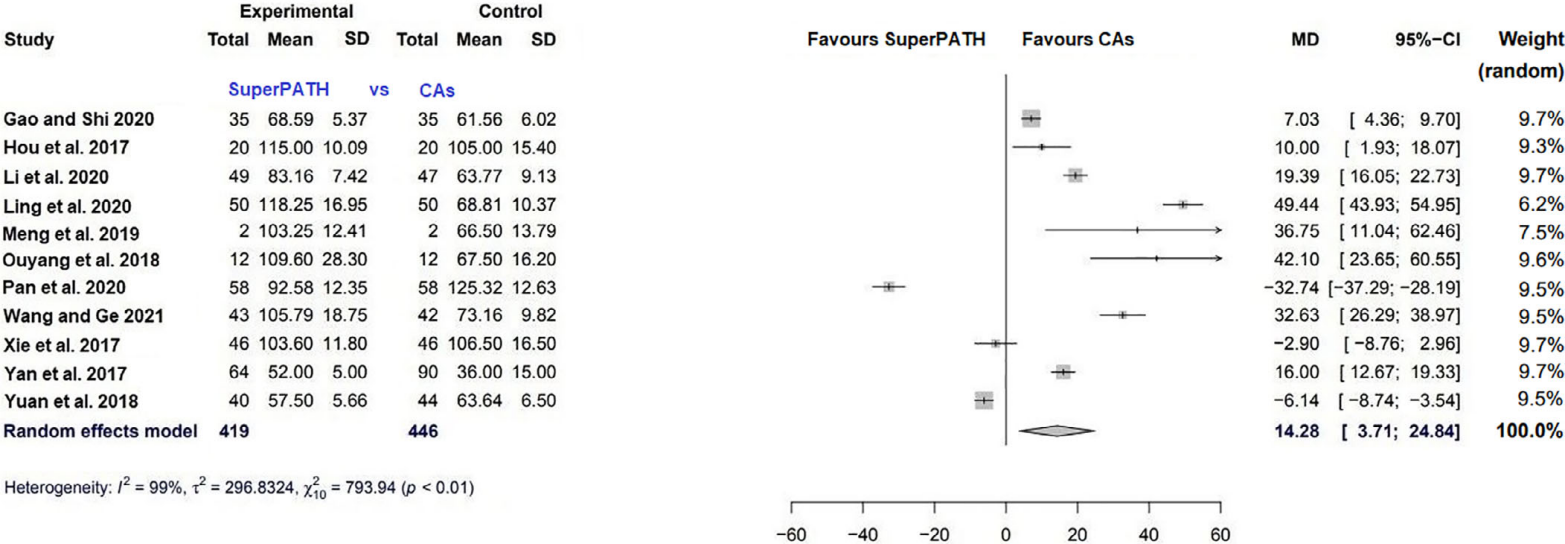
Forest plot of the secondary outcome: operation time in min. CAs, conventional approaches; SD, standard deviation; MD, mean difference; CI, confidence interval

Data on 865 patients were pooled from 11 RCTs (*I* ^2^ = 99%, *P* < 0.01, Fig. 6). The operation time of SuperPATH was 14.3 min longer than the operation time of CAs (MD = 14.3, 95% CI 3.7–24.8).

##### Incision Length

**Fig. 7:**
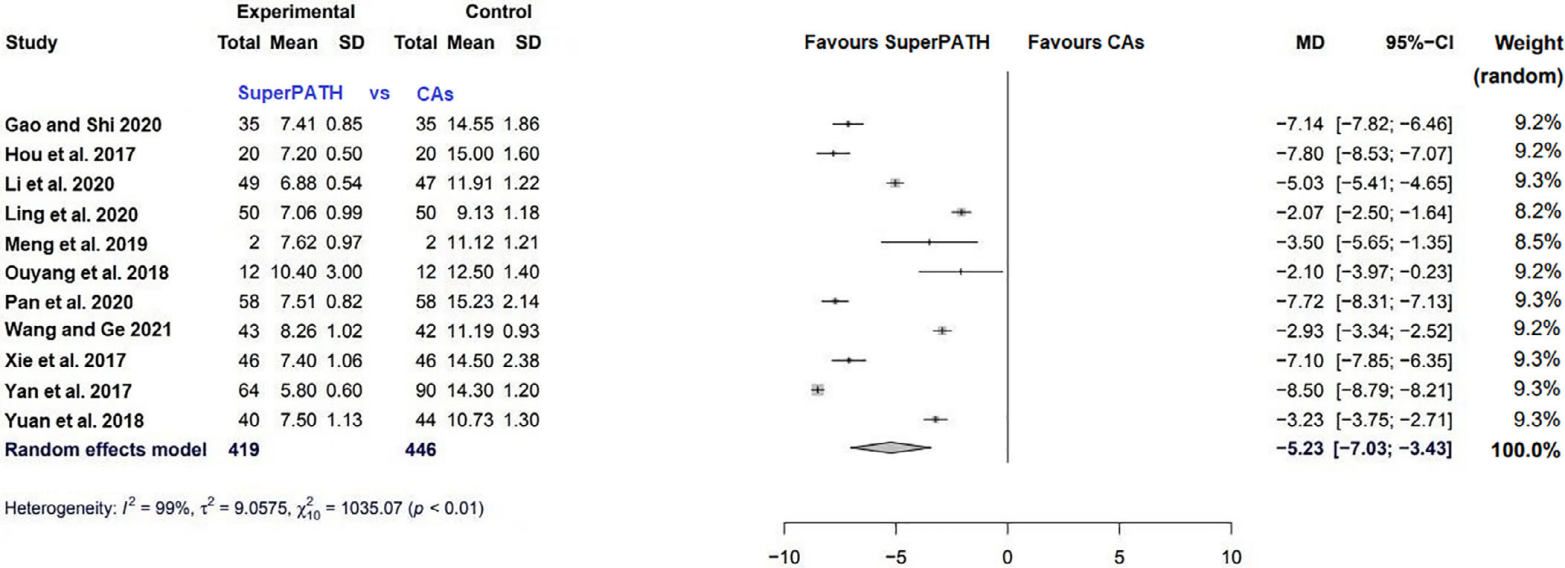
Forest plot of the secondary outcome: incision length in cm. CAs, conventional approaches; SD, standard deviation; MD, mean difference; CI, confidence interval

Data on 865 patients were pooled from 11 RCTs (*I* ^2^ = 99%, *P* < 0.01, Fig. 7). The incision length of SuperPATH was 5.2 cm shorter than the incision length of CAs (MD = −5.2, 95% CI −7.0 to −3.4).

##### Acetabular Cup Inclination Angle

**Fig. 8:**
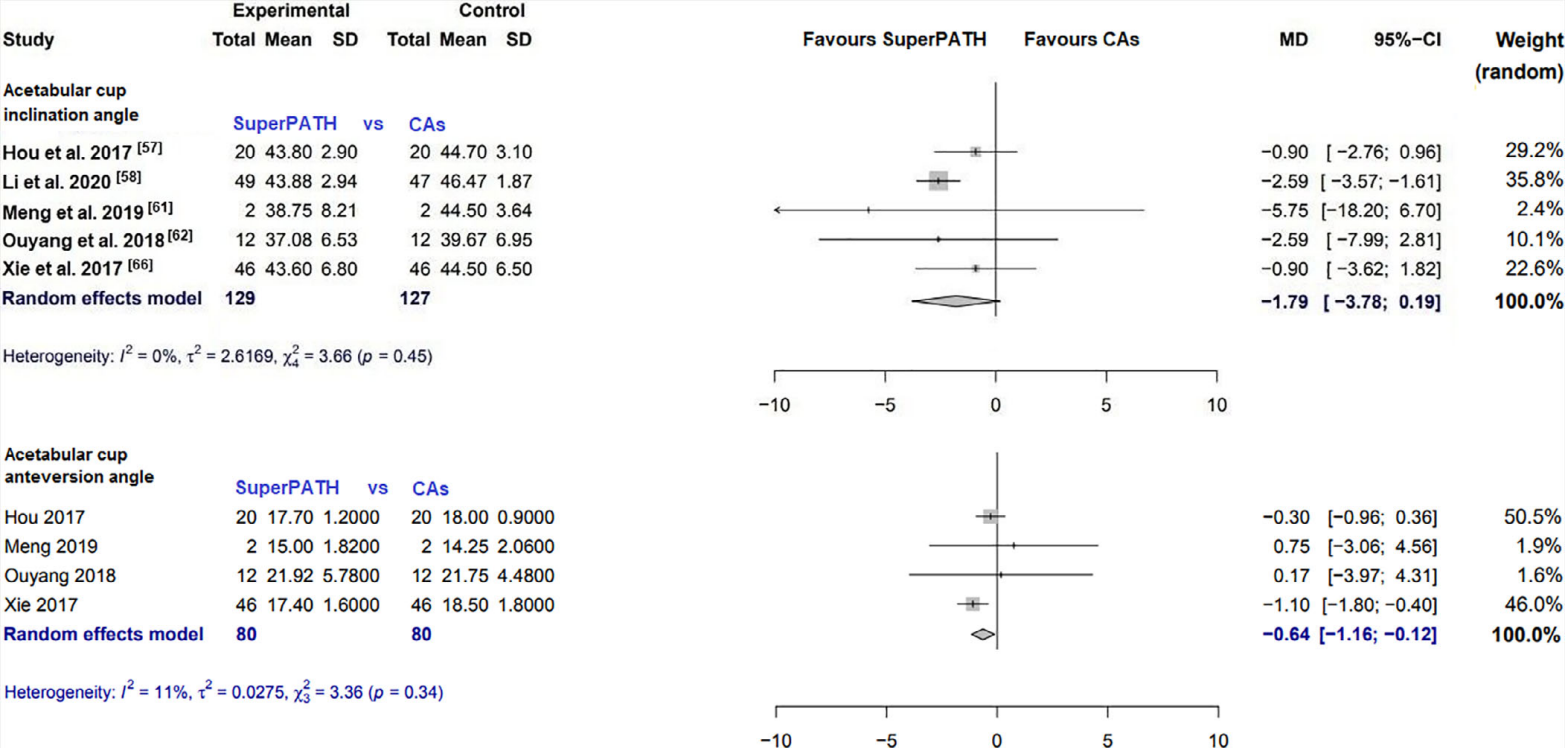
Forest plot of the secondary outcome: acetabular cup inclination and anteversionangles in degrees. CAs, conventional approaches; SD, standard deviation; MD, mean difference; CI, confidence interval

Data on 256 patients were pooled from five RCTs (*I* ^2^ = 0%, *P* = 0.45, Fig. 8). There was no difference in acetabular cup inclination angle (MD = −1.8, 95% CI −3.8–0.2).

##### Acetabular Cup Anteversion Angle

Data on 160 patients were pooled from four RCTs (*I* ^2^ = 11%, *P* = 0.34, Fig. 8). The acetabular cup anteversion angle of SuperPATH was 0.6° lower than the acetabular cup anteversion angle of CAs (MD = −0.6, 95% CI −1.2 to −0.1).

**Fig. 9:**
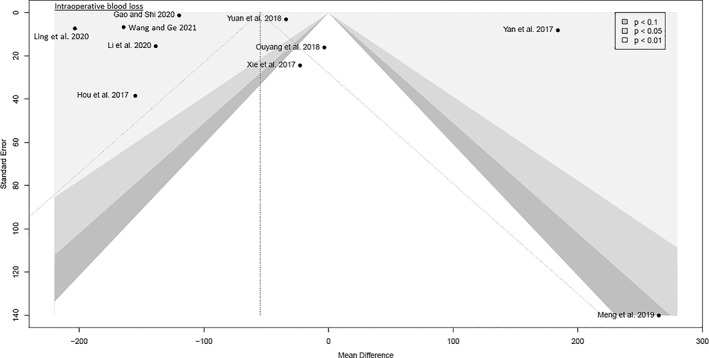
Funnel plot of the primary outcome: intraoperative blood loss

**Fig. 10:**
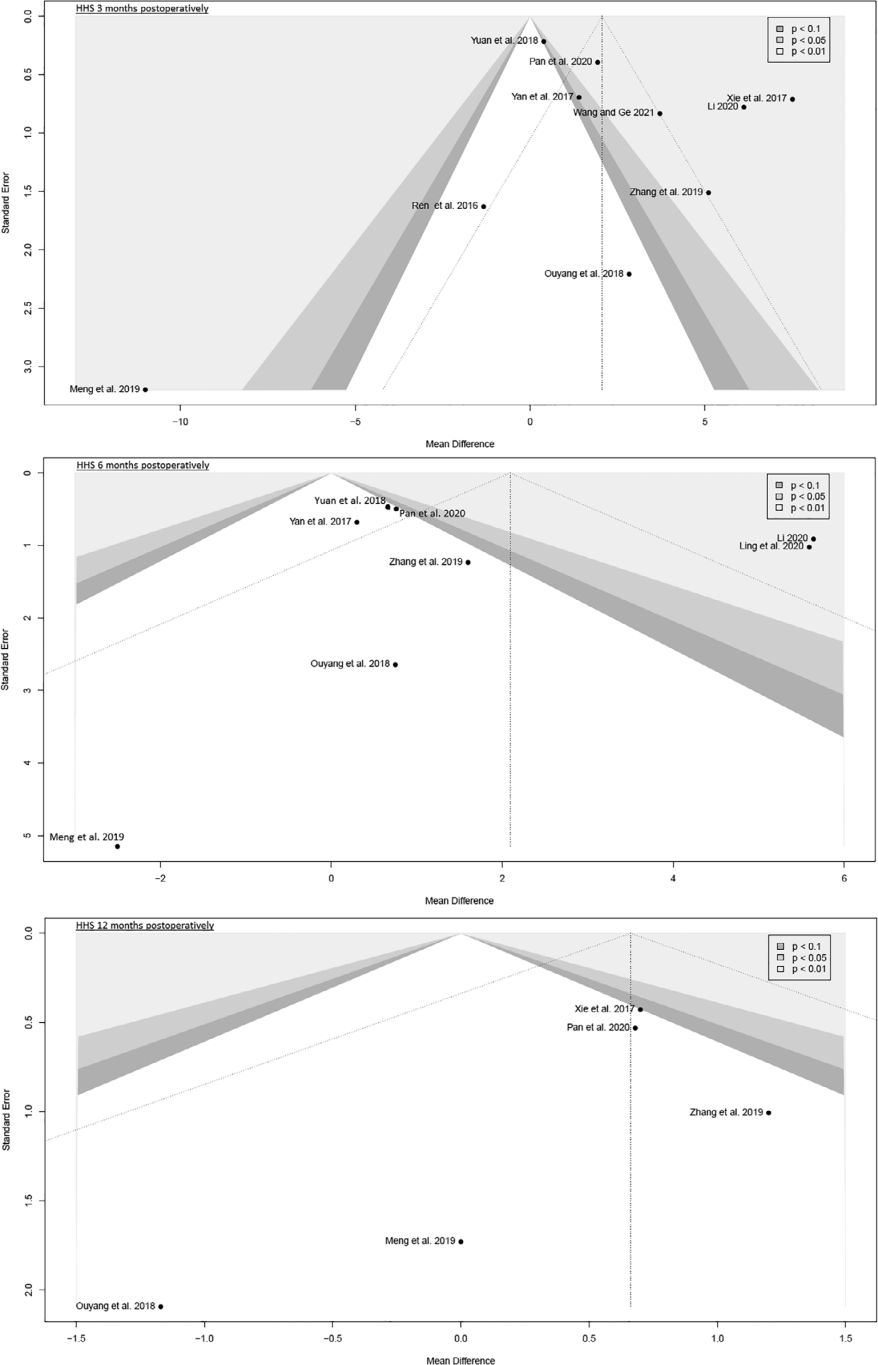
Funnel plot of the primary outcome: HHS 3, 6 and 12 months postoperatively. HHS: Harris hip score

**Fig. 11:**
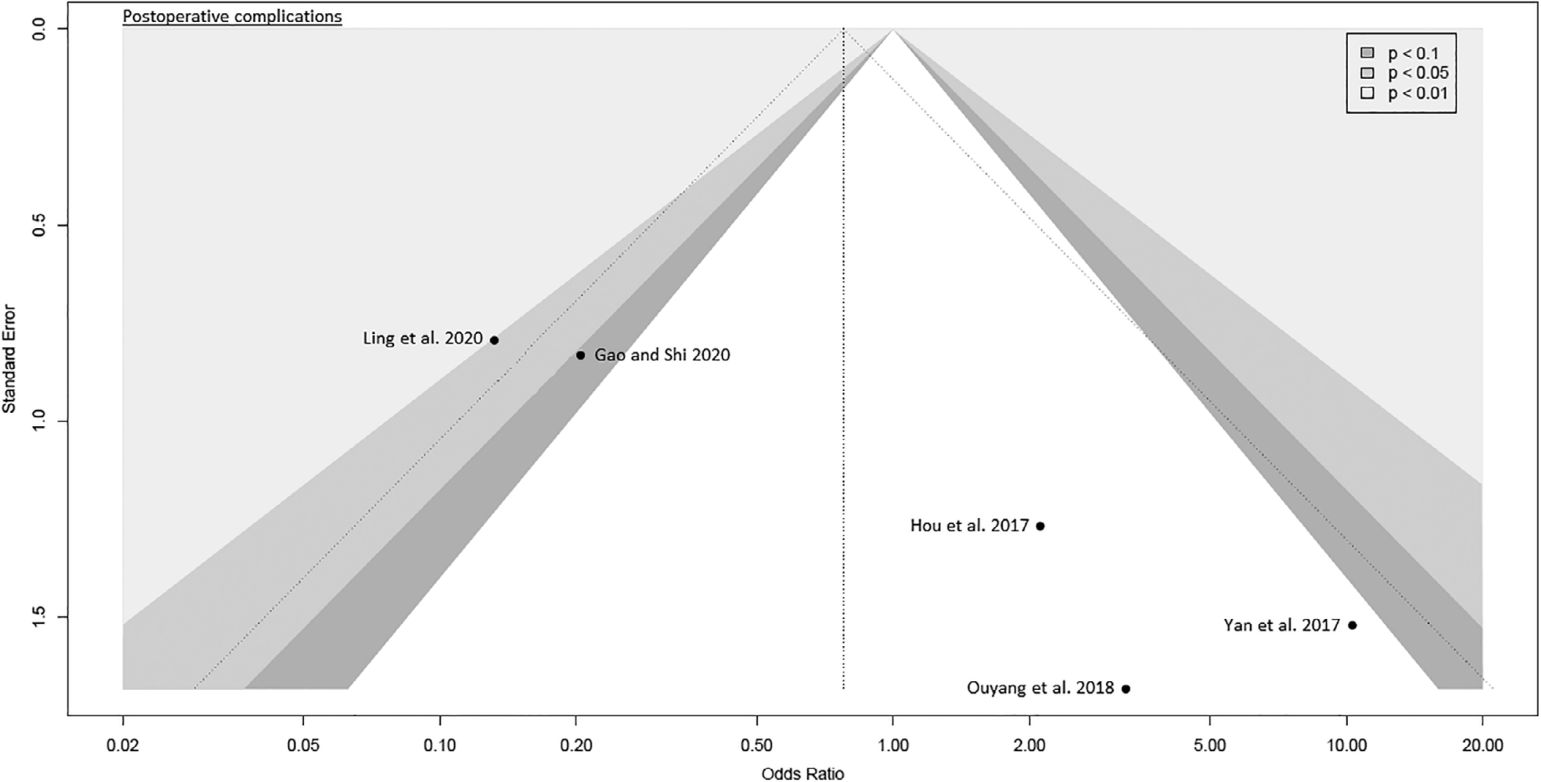
Funnel plot of the primary outcome: postoperative complication rates

**Fig. 12:**
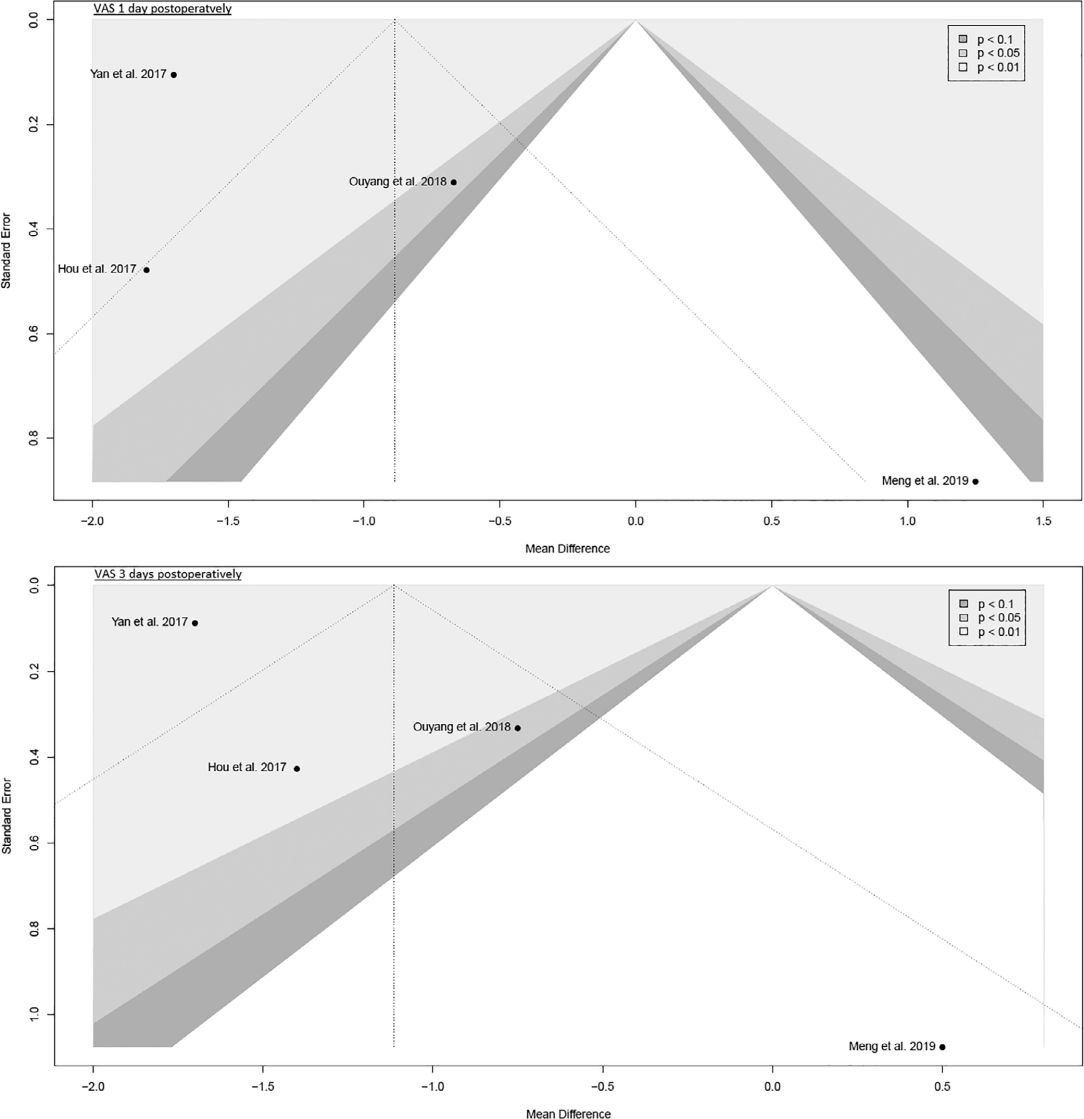
Funnel plot of the secondary outcome: pain VAS 1 day and 3 days postoperatively. VAS: visual analog scale

**Fig. 13:**
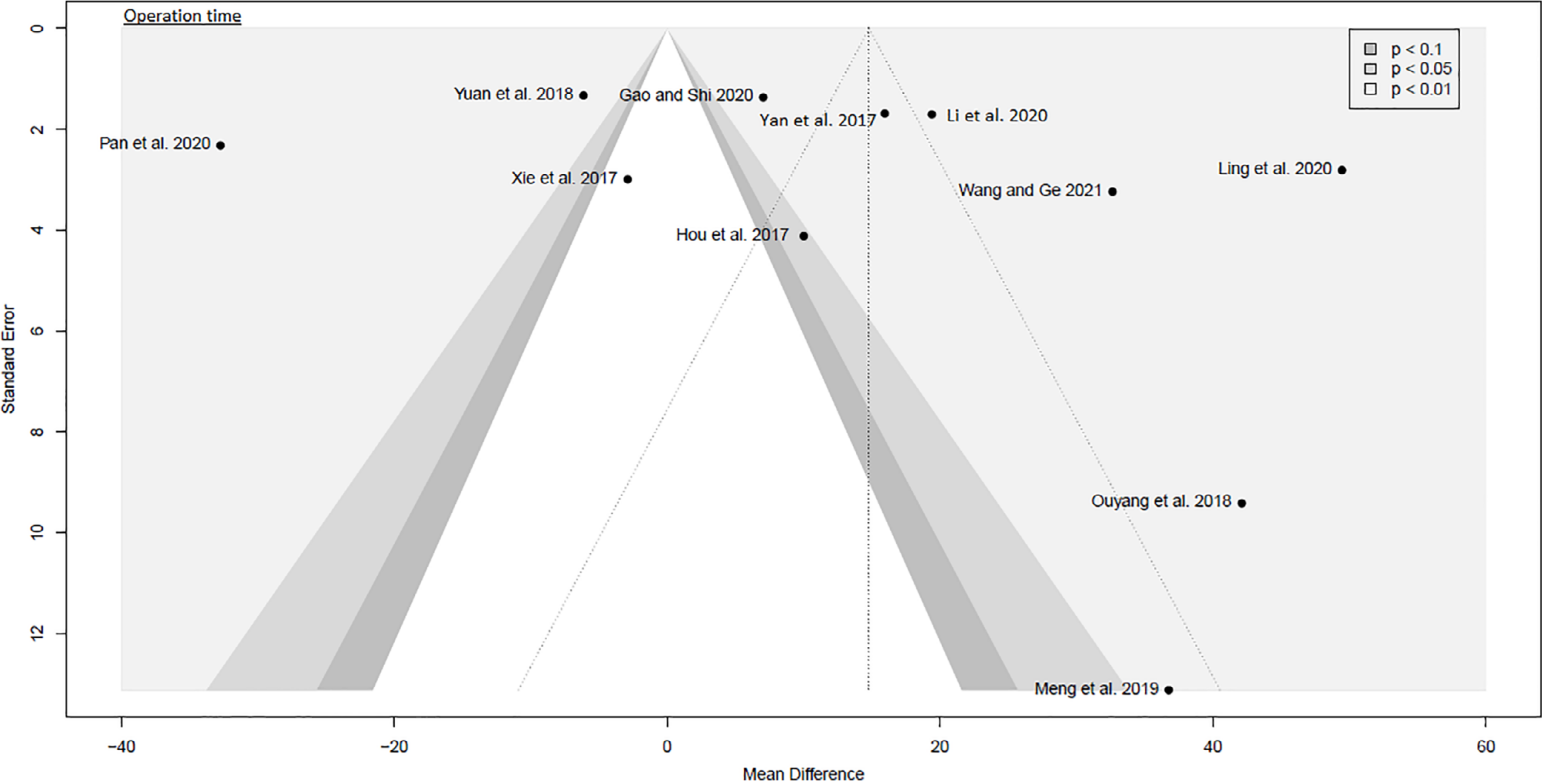
Funnel plot of the secondary outcome: operation time

**Fig. 14:**
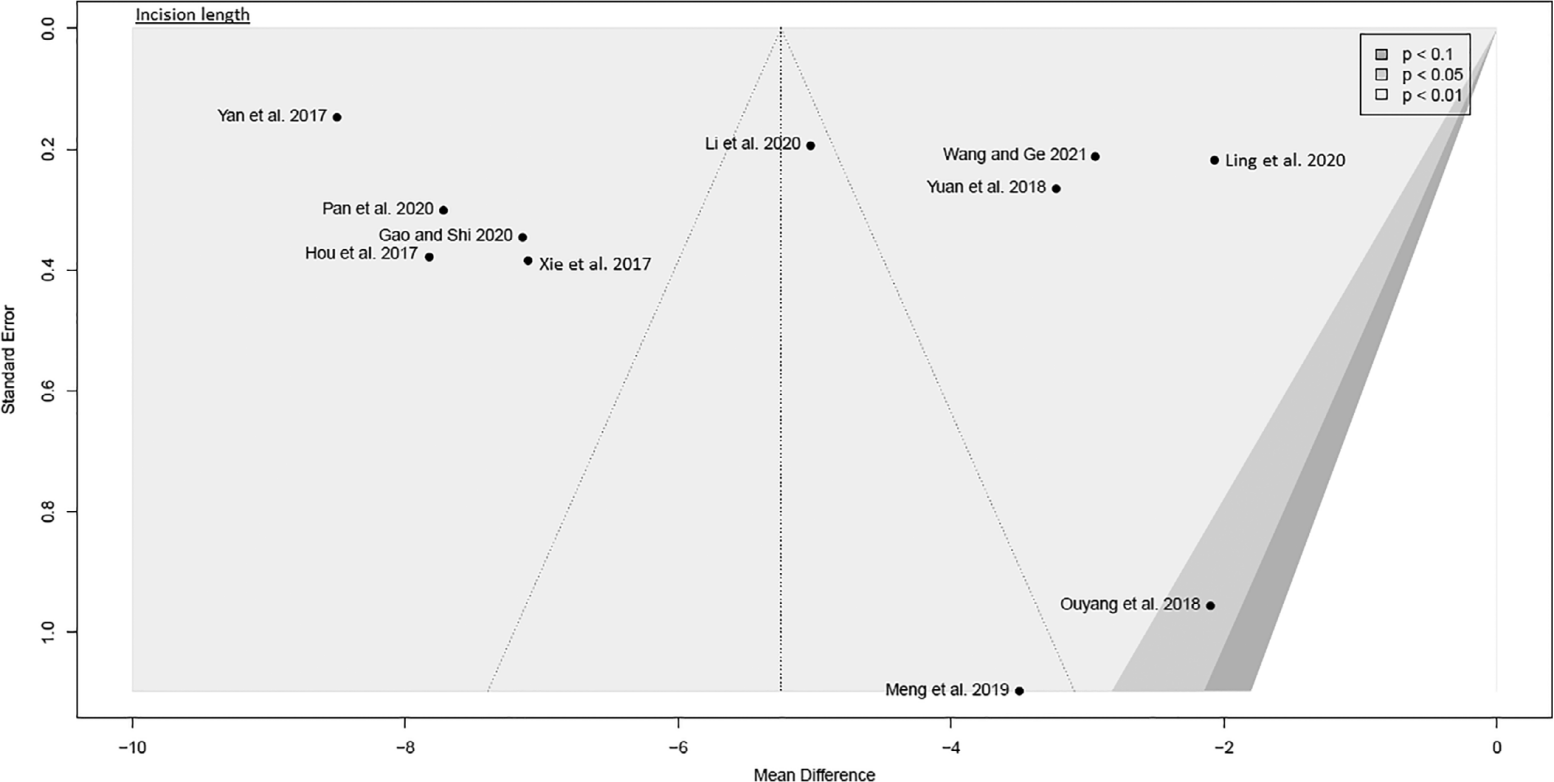
Funnel plot of the secondary outcome: incision length

**Fig. 15:**
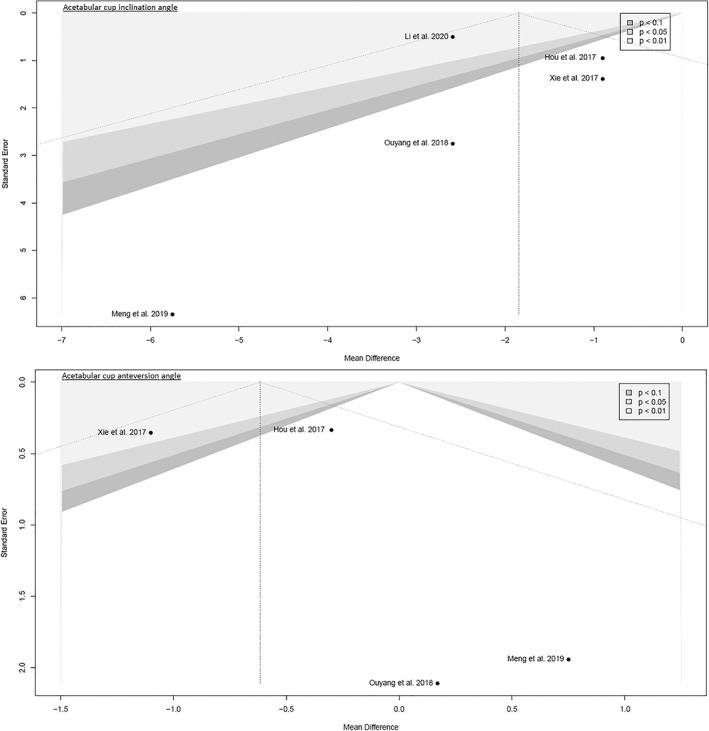
Funnel plot of the secondary outcome: acetabular cup inclination and anteversion angles

The funnel plots were broadly symmetrical, indicating minimal to moderate publication bias (Figs 9, 10, 11, 12, 13, 14, 15). Forrest plots including both fixed and random effects models are given in supplement.

## Discussion

### Main and New Findings

Fourteen RCTs with 1021 patients were included in this meta‐analysis (Meta‐SuCAs‐2). Two out of 14 RCTs were rated with a low risk of bias^16^, ^19^, nine RCTs with a moderate risk of bias^14^, ^15^, ^20^, ^21^, ^23^, ^24^, ^25^, ^26^, ^27^, and three RCTs with a high risk of bias^17^, ^18^, ^22^. The level of evidence was assessed according to GRADE recommendations (Table 4). The funnel plots were broadly symmetrical, indicating minimal to moderate publication bias (Figs 9, 10, 11, 12, 13, 14, 15). In general, Meta‐SuCAs‐2 indicated that THA through SuperPATH was superior to THA through CAs regarding the investigated primary and secondary outcomes. SuperPATH showed better results on decreasing intraoperative blood loss and on increasing postoperative HHS in THA compared to CAs. Both approaches did not differ in postoperative complication rates. SuperPATH showed better results on decreasing early postoperative VAS and incision length. Both approaches showed sufficient results in acetabular cup positioning. THA through SuperPATH had a longer operation time than CAs.

Meta‐SuCAs‐2 was a continuation and update of the first meta‐analysis in English language on THA through SuperPATH *vs*. CAs^6^, which was published in 2020 by Ramadanov *et al*. By including further RCTs and increasing the overall sample size, a clearer conclusion was drawn based on the results of Meta‐SuCAs‐2. Meta‐SuCAs‐2 used high‐quality statistical methods as it was estimated with both fixed and random effects models and thus provided higher informative value. Last but not least, Meta‐SuCAs‐2 is an attempt on the part of the author (NR) to correct a misinterpretation from his first meta‐analysis on THA through SuperPATH *vs*. CAs.^6^

### Comparison with Related Meta‐Analyses

The results of Meta‐SuCAs‐2 were similar to the findings of recent literature. The 2018 meta‐analysis by Li *et al*.^4^ with eight RCTs and 483 patients showed overall better results for hip arthroplasty through SuperPATH. SuperPATH had a shorter incision length, a lower intraoperative blood loss, and early pain VAS 1, 3, and 7 days postoperatively than CAs. SuperPATH showed a higher HHS 3 months postoperatively. The subsequent HHS 6 months postoperatively showed no difference. The 2018 meta‐analysis by Li *et al*. did not find differences in the acetabular cup positioning. SuperPATH had a longer operation time than CAs. The study by Li *et al*.^4^ had severe limitations; it stated it had included eight randomized controlled trials (RCTs). Unfortunately, two of those RCTs claimed to be retrospective^28^, ^29^. A third one of the included RCTs in this Chinese meta‐analysis^4^ claimed to be prospective in Chinese, but retrospective in its English abstract^30^. Nevertheless, this RCT was not categorized as “randomized”^30^. In addition, the confounding of hemiarthroplasty^31^ and THA, as well as conventional and mini‐incision approaches^32^, poses another severe limitation to this meta‐analysis^4^.

The 2018 meta‐analysis by Sun *et al*.^5^ with four studies (three RCTs and one observational study) and 266 patients also showed almost indifferent results for hip arthroplasty through SuperPATH compared to CAs. SuperPATH had a shorter incision length than CAs. This meta‐analysis showed equal results between SuperPATH and CAs concerning operation time, intraoperative blood loss, postoperative HHS, and acetabular cup inclination angles. The 2018 meta‐analysis by Sun^5^ had a very small sample size. It included only four trials, one of which being an observational study.

The 2020 meta‐analysis by Ramadanov *et al*.,^6^ with 12 RCTs and 726 patients, also showed overall better results for hip arthroplasty through SuperPATH. This meta‐analysis^6^ compared short‐term outcomes of SuperPATH and CAs in both THA and hemiarthroplasty. The results of the THA‐subgroup did not differ from the overall results of the THA/hemiarthroplasty group in a clinically relevant manner. This meta‐analysis^6^ showed that SuperPATH reduced incision length, pain VAS 7 days postoperatively. SuperPATH had a higher HHS 7 days postoperatively than CAs. The two approaches did not differ in acetabular cup positioning angles, intra‐ and postoperative blood loss, hospitalization period, and postoperative complications. SuperPATH showed a longer operation time than hip replacement through CAs. However, this meta‐analysis^6^ showed high heterogeneity in many outcome parameters, which limited the conclusions. Furthermore, there was a severe error in interpreting the results. It found a difference in Harris hip score (HHS) 7 days postoperatively (MD = 10.24, 95% CI 0.27–20.21). In the related forest plot, the designations on the graphic “FavoursSuperPATH group” and “Favours conventional approach group” were swapped. Looking at this graphic, it appears that the CA group had a higher HHS. In fact, the SuperPATH group had a 10.2 points higher HHS 7 days postoperatively as described in the text of the result section of the same meta‐analysis^6^. This resulted in another error in the result section of the abstract of this meta‐analysis^6^. There it was written that the HHS of the SuperPATH group was reduced compared to the CA group. In fact, it was increased as stated in the discussion and conclusion section of the same meta‐analysis^6^.

A recently published 2021 meta‐analysis by Ge *et al*.^7^ with six studies and 526 patients showed overall better results for hip arthroplasty through SuperPATH. SuperPATH had a shorter incision length, decreased blood transfusion rate, a lower pain VAS 1 week postoperatively, and higher HHS than the conventional group. However, there was no difference in subsequent VAS, operation time, and in the acetabular abduction angle. An important limitation of this meta‐analysis is the confounding factor of hemiarthroplasty^33^ and total hip arthroplasty (THA), and the moderate sample size of 526 patients.

### Primary Outcomes

#### Intraoperative Blood Loss

Intraoperative blood loss was defined as the total amount of blood from the suction device. It reflects the severity of intraoperative trauma. The median intraoperative blood loss was 132 ml and ranged from 89 to 1108 ml for SuperPATH. The median intraoperative blood loss was 209 ml and ranged from 142 to 844 ml for CAs. SuperPATH had a 61 ml lower intraoperative blood loss than CAs (MD = −61.4, 95% CI −119.1 to −3.8). This outcome parameter had considerable heterogeneity (*I* ^2^ = 100%), with 2 RCTs^19^, ^25^ showing better results for CAs and 8 RCTs^14^, ^15^, ^16^, ^18^, ^20^, ^23^, ^24^, ^26^ showing better results for SuperPATH. In general, the literature shows the superiority of mini‐incision approaches in reducing blood loss compared to standard approaches^34^. Since SuperPATH is a minimally‐invasive approach, an explanation for the lower intraoperative blood loss can be found in the lower tissue traumatization. The utilization of tranexamic acid and intraoperative active warming are further known factors influencing blood loss in THA besides approaches to the hip joint^35^, ^36^, ^37^.

#### Harris Hip Score

The HHS was developed for the assessment of the results of hip surgery^38^. The score collects points from the assessment of four aspects: pain, function, degree of deformity, and range of motion of the hip. The higher the added score, the better the results, providing a range of added scores from 0 to 100 points^38^. The median HHS 3 months postoperatively was 85 points and ranged from 72.3 to 93.3 points for SuperPATH. The median HHS 3 months postoperatively was 79.5 points and ranged from 78 to 90.3 points for CAs. The median HHS 6 months postoperatively was 92 points and ranged from 84.3 to 95.8 points for SuperPATH. The median HHS 6 months postoperatively was 88.5 points and ranged from 79.7 to 92.9 points for CAs. The median HHS 12 months postoperatively was 92 points and ranged from 85.6 to 94.7 points for SuperPATH. The median HHS 12 months postoperatively was 91 points and ranged from 86.8 to 94 points. The HHS 3 and 6 months postoperatively had considerable heterogeneity (*I* ^2^ = 95%; *I* ^2^ = 85%), the HHS 12 months postoperatively had very low heterogeneity (*I* ^2^ = 0%).With a few exceptions, all RCTs showed better results for SuperPATH in HHS than for CAs. One RCT^19^ was noticeable, showing poorer results for SuperPATH in the HHS 3 and 6 months postoperatively and showing indifferent results in the HHS 12 months postoperatively. The HHS 3 months postoperatively of SuperPATH was 2.4 points higher than the HHS of CAs (MD = 2.4, 95% CI 0.6–4.2), the HHS 6 months postoperatively of SuperPATH was 2.1 points higher than the HHS of CAs (MD = 2.1, 95% CI 0.6–3.6), and the HHS 12 months postoperatively of SuperPATH was 0.7 points higher than the HHS of CAs (MD = 0.7, 95% CI 0.1–1.3). This shows that the advantage of SuperPATH lies particularly in the achievement of good early functionality since the HHS difference between SuperPATH and CAs levels out in the postoperative course. This is to be recognized as a decisive advantage of SuperPATH since ultimately improving the functionality of the hip joint is the primary treatment goal of the THA in patients with osteoarthritis. One possible explanation for this advantage is the operation in a tissue‐sparing plane. The bad impact of soft tissue traumatizationon function is well known in surgery.

#### Postoperative Complications

Postoperative complications are a very important outcome parameter for every surgical technique and approach. THA through SuperPATH showed 10 events of postoperative complications out of 181 cases, THA through CAs showed 21 events of postoperative complications out of 207 cases. This outcome parameter had moderate heterogeneity (*I* ^2^ = 62%), with 2 RCTs^14^, ^18^ showing better results for SuperPATH and 3 RCTs^15^, ^20^, ^25^ showing better results for CAs. All in all, SuperPATH and CAs did not differ in postoperative complication rates (OR = 0.7, 95% CI 0.2–3.3). However, our sample size did not allow us to distinguish between different types of postoperative complications and to meta‐analyze them individually.

### Secondary Outcomes

#### Pain VAS

The pain VAS is an instrument for measuring pain intensity, providing a score range from 0 to 10 points^39^, ^40^. The median pain VAS 1 day postoperatively was 4 points and ranged from 3.1 to 8.3 points for SuperPATH. The median pain VAS 1 day postoperatively was 6 points and ranged from 4.2 to 7 points for CAs. SuperPATH had a 1 point lower pain VAS 1 day postoperatively than CAs (MD = −1.0, 95% CI −1.8 to −0.2). The median pain VAS 3 days postoperatively was 2 points and ranged from 1.5 to 7 points for SuperPATH. The median pain VAS 3 days postoperatively was 4 points and ranged and from 2.9 to 6.5 points for CAs. SuperPATH had a 1.2 points lower pain VAS 3 days postoperatively than CAs (MD = −1.2, 95% CI −1.8 to −0.5). Pain VAS 1 day postoperatively had a considerable heterogeneity (*I* ^2^ = 85%), pain VAS 3 days postoperatively had a moderate heterogeneity (*I* ^2^ = 75%), with one RCT^19^ showing better results for CAs and three RCTs^15^, ^20^, ^25^ showing better results for SuperPATH. Postoperative pain is an expected but yet undesirable side effect of all surgical interventions. It has a strong influence on the overall well‐being of the patient. The lower early pain VAS postoperatively is an important advantage of SuperPATH. Again, the possible explanation for this advantage is the lower tissue traumatization in THA through SuperPATH. A recent NMAs found that the good ways to reduce early THA pain is the spinal anesthesia and lumbar plexus block, followed by the local infiltration analgesia, followed by the opioid consumption^41^, ^42^.

#### Operation Time

The operation time was defined as the period of time from the beginning of skin incision to suture. The median operation time in Meta‐SuCAs‐2 was 63 min and ranged from 52 to 118 min for SuperPATH. The median operation time was 49 min and ranged from 36 to 125 min for CAs. This outcome parameter had considerable heterogeneity (*I* ^2^ = 99%). SuperPATH had a 14 min longer operation time (MD = 14.3, 95% CI 3.7 to 24.8). This was the only disadvantage of THA through SuperPATH found. Prolonged operative times are known to be associated with increased rates of superficial infections and perioperative complications^43^, ^44^. In a 2019 analysis of 89,802 cases of THA the authors found an operation time of approximately 80 min is associated with a lower risk of perioperative complications^44^. SuperPATH is a novel approach with a prolonged learning curve for operating surgeons^45^. Therefore, this approach probably has potential for a shorter operation time with increasing proficiency.

#### Incision Length

The incision length was measured on a graduated scale. It reflects the severity of intraoperative trauma. Since the SuperPATH is a 2‐incision approach, it remained unclear whether the included RCTs reported the added incision length or the length of the larger incision, ignoring the smaller additional incision. The median incision length was 7 cm and ranged from 5.8 to 10.4 cm for SuperPATH. The median incision length was 13 cm and ranged from 9.1 to 15.2 cm for CAs. This outcome parameter had considerable heterogeneity (*I* ^2^ = 99%). SuperPATH had a 5.2 cm shorter incision length than CAs (MD = −5.2, 95% CI −7.0 to −3.4). The literature is inconclusive about the importance of incision length^46^, ^47^. Incision length is associated with patient weight, height, and gender. Large and obese patients, as well as female patients, are more likely to receive longer incisions in mini‐incision THA^48^.

#### Acetabular Cup Positioning

The acetabular cup anteversion angle and the inclination angle in degrees have ideal values for positioning: anteversion angle from 10° to 25° and inclination angle from 40° to 50°^49^. Especially, the ideal acetabular cup anteversion is of great importance, since an angle too large often leads to posterior impingement, resulting in anterior dislocation, and an angle too small leads to posterior dislocation. The median acetabular cup inclination angle was 44° and ranged from 37.1° to 43.9° for SuperPATH. The median acetabular cup inclination angle was 45° and ranged from 39.6° to 47.1° for CAs. The median acetabular cup anteversion angle was 17.5° and ranged from 15° to 21.9° for SuperPATH. The median acetabular cup anteversion angle was 18° and ranged from 14.3° to 21.8° for CAs. Acetabular cup positioning through SuperPATH and Cas fit relatively well within the stated normal values. It is important to know that the acetabular cup anteversion angle is a very questionable outcome parameter since it was measured in almost every study with conventional radiographs. The acetabular cup anteversion angle can only be measured reliably with a CT scan^50^. The acetabular cup anteversion angle and the inclination angle had very low heterogeneity (*I* ^2^ = 0%; *I* ^2^ = 11%).

When interpreting the results, however, the question arises whether the outcome differences were clinically important. Some of the measured outcomes in Meta‐SuCAs‐2 showed statistically significant, but clinically minor differences. Therefore, it is questionable whether these results justify a change in the surgical approach.

### Study Limitations

Meta‐SuCAs‐2 had several limitations: due to insufficient data, important outcome parameters were not meta‐analyzed. Confounding factors like the surgeon operating skills, the utilization of tranexamic acid and anticoagulants, bone cement, or the types of implants for hip replacement probably influenced the results. Part of the RCTs did not give any information on what exact hip pathology was treated with THA. It remained unclear whether the included RCTs reported the added incision length or the length of the larger incision in THA through SuperPATH, ignoring the smaller additional incision. In some outcome parameters, considerable heterogeneity between individual studies was observed, which might bias the results.

## Conclusion

The overall findings of Meta‐SuCAs‐2 suggested that the short‐term outcomes of THA through SuperPATH were superior CAs in all measured surgical and functional outcomes besides operation time. In primary outcome, SuperPATH had a lower intraoperative blood loss and a higher HHS. Both approaches did not differ in postoperative complications. In secondary outcome, SuperPATH had a lower pain VAS and a shorter incision length. Both approaches showed sufficient results in acetabular cup positioning. CAs had a shorter operation time than SuperPATH.

## Conflict of interest

Not applicable.

## Authors' contributions

NR conducted the whole work with minor help from colleagues (RK, KL, SB).

## Data Availability

The data are available from the corresponding author upon reasonable request.
